# Missed Opportunities for HIV Testing in Hospitalised Adults in Türkiye: Indicator Conditions and Testing Coverage in a National Multicentre Point-Prevalence Survey (HIV-ICs-TR)

**DOI:** 10.1038/s41598-026-54294-6

**Published:** 2026-05-30

**Authors:** Yeliz Çiçek, Mustafa Kemal Çelen, Hüsnü Pullukçu, Seyit Ali Büyüktuna, Fatma Kesmez Can, Damla Ertürk, Buket Erturk Sengel, Hanife Nur Karakoc Parlayan, İlknur Esen Yıldız, Kemalettin Özden, Handan Alay, Ayşe Albayrak, Duygu Aran Seçkin, Galip Erdoğan, Tajdin İrdem, Betül Seçen Gülçek, Kübra Okul, Derya Kaya, Cansu Tol, Mehmet Buğra Özkara, Meltem Ceylan, Oğuzhan Acet, Caner Öksüz, Rıdvan Karaarslan, Zülal Peri Yoldaş Aslanoğlu, Esra İrem Dağcı, Süheyla Kömür, Sümeyra Demir, Ömer Faruk Yardımcı, Gökçe Melis Çolak, Diana Zakaradze, Esma Demir Kalyoncu, Betul Ocak, Gürdal Yılmaz, Ahmet Turan Koç, Adem Kaya, Maşite Nur Özdemir, Gülter Öncü Kurutaş, Tuba İlgar, Aybegüm Özşahin, Sudem Mahmutoğlu-Çolak, Kenan Beşbaş, Yeşim Taşova

**Affiliations:** 1 Epidemiology Doctorate Program, Graduate School of Health Sciences, Istanbul Medipol University, Istanbul, Türkiye; 2 Department of Infectious Diseases and Clinical Microbiology, Dicle University, Diyarbakır, Türkiye; 3 Department of Infectious Diseases and Clinical Microbiology, Faculty of Medicine, Ege University, Izmir, Türkiye; 4 Department of Infectious Diseases and Clinical Microbiology, Sivas Cumhuriyet University, Sivas, Türkiye; 5 Department of Infectious Diseases and Clinical Microbiology, Atatürk University School of Medicine, Erzurum, Türkiye; 6 Department of Infectious Diseases and Clinical Microbiology, Faculty of Medicine, Çukurova University, Adana, Türkiye; 7 Department of Infectious Diseases and Clinical Microbiology, Marmara University Pendik Training and Research Hospital, Istanbul, Türkiye; 8 Department of Infectious Diseases and Clinical Microbiology, Faculty of Medicine, Karadeniz Technical University, 61080 Trabzon, Türkiye; 9 Department of Infectious Diseases and Clinical Microbiology, Recep Tayyip Erdoğan Training and Research Hospital, Rize, Türkiye

**Keywords:** HIV testing, Inpatients, Early diagnosis, Missed diagnosis, HIV ınfections, Türkiye, Diseases, Health care, Medical research, Risk factors

## Abstract

To quantify the prevalence of HIV testing indications among hospitalised adults in Türkiye, assess HIV testing coverage when clinically indicated, and identify missed diagnostic opportunities in a national multicentre point-prevalence survey. We conducted a national multicentre cross-sectional point-prevalence survey across tertiary referral hospitals from all seven geographical regions of Türkiye. Adult inpatients present at 08:00 on a predefined index day were assessed for predefined HIV testing indications, including AIDS-defining conditions, indicator conditions associated with an expected undiagnosed HIV prevalence ≥ 0.1%, and conditions in which unrecognised HIV could substantially affect clinical management. Among 3,425 hospitalised adults without known HIV infection, 699 (20.4%) had at least one documented HIV testing indication. HIV testing was requested by the primary clinical team in 424/699 patients (60.7%). Testing was lowest in patients with indicator conditions associated with an expected undiagnosed HIV prevalence ≥ 0.1%, and these conditions were independently associated with higher odds of non-performance of HIV testing by the primary clinical team than AIDS-defining conditions (corresponding adjusted odds ratio [aOR] 3.05, 95% confidence interval [CI] 1.67–5.81). In centres with a specialised HIV outpatient clinic, the odds of non-performance of HIV testing by the primary clinical team were independently lower among patients with an appropriate testing indication (corresponding aOR 0.64, 95% CI 0.43–0.95). Following study-team facilitation, overall testing coverage increased to 677/699 (96.9%). Three reactive screening results were identified, including two confirmed new HIV diagnoses. HIV testing indications were common among hospitalised adults in Türkiye, but non-performance of HIV testing by the primary clinical team remained frequent, particularly for non-AIDS indicator conditions. Strengthening indicator-condition-guided testing through structured clinical pathways and institutional HIV expertise may reduce non-performance and support earlier HIV diagnosis.

**Supplementary Information:**

The online version contains supplementary material available at 10.1038/s41598-026-54294-6.

## Introduction

Human immunodeficiency virus (HIV) infection remains a major global public health challenge despite substantial advances in antiretroviral therapy (ART). By the end of 2024, an estimated 40.8 million people were living with HIV globally, with approximately 1.3 million new infections and 630,000 AIDS-related deaths estimated during the same year^1^. Persistent gaps in timely HIV diagnosis continue to contribute to preventable morbidity and mortality, and European hospital-based testing interventions have been shown to increase HIV testing and case detection, underscoring hospitals as a critical point of contact for HIV diagnosis^2^.

Within the WHO European Region, 105,922 new HIV diagnoses were reported in 2024, while the number of people living with undiagnosed HIV has continued to increase. Late HIV diagnosis remains a major surveillance and clinical concern and is commonly classified using CD4 T-cell thresholds and AIDS-defining events, with updated consensus recommendations further specifying the classification of recent infections and previously diagnosed individuals in surveillance and research settings^3–6^. Late HIV diagnosis is associated with higher rates of hospitalisation, opportunistic infections, early mortality, and continued HIV transmission^7^. These findings underscore the clinical and public health importance of strengthening timely HIV testing strategies across healthcare settings.

National surveillance data indicate a sustained increase in notified HIV diagnoses in Türkiye. From 1985 to 10 November 2025, a total of 54,472 confirmed HIV diagnoses and 2,629 AIDS cases were reported^8, 9^. In the most recent complete calendar year, 2024, 7,135 new HIV diagnoses and 158 AIDS cases were notified^8^, 9. Although Türkiye remains a non-generalised HIV epidemic setting, these national notification data indicate an increasing number of reported HIV diagnoses and underscore the need to strengthen early diagnosis and timely linkage to care across healthcare settings. This concern is reinforced by evidence from Türkiye showing substantial missed opportunities for earlier HIV diagnosis, with HIV testing not offered in 77.9% of visits where a testing indication was present^10^. In parallel, modelling analyses grounded in epidemiological data from Türkiye suggest that, if current epidemic dynamics persist, the national HIV burden could increase substantially by 2030, with a large proportion of people living with HIV remaining undiagnosed^11^. Together, these findings highlight the importance of earlier HIV testing strategies and timely recognition of clinical presentations that should prompt diagnostic evaluation.

Hospitals constitute a central platform for HIV case identification, and testing strategies in healthcare settings should be aligned with local epidemiology. In higher-prevalence settings, routine opt-out testing in emergency departments and inpatient wards has been shown to be cost-effective and to improve case detection^12^. UK HIV testing guidance operationally defines high-prevalence areas as those with diagnosed HIV prevalence of 2–5 per 1,000 adults aged 15–59 years, and extremely high-prevalence areas as those with > 5 per 1,000; these thresholds are used to support broader routine or opt-out HIV testing in selected healthcare settings^13^. In non-generalised HIV epidemic settings such as Türkiye, indicator-condition–guided testing strategies are generally considered more efficient, particularly when the expected undiagnosed HIV prevalence in the target clinical population is ≥ 0.1% ^14, 15^. Evidence from multicentre European studies, including HIDES I and HIDES II, has shown that several clinical presentations are associated with undiagnosed HIV prevalence well above this threshold^16, 17^. However, real-world implementation remains suboptimal, with testing coverage frequently falling below recommended levels among clinically eligible patients^18, 19^.

On 30 June 2025, the Republic of Türkiye Ministry of Health, General Directorate of Public Health, issued an official institutional communication to provincial health directorates regarding the implementation of HIV diagnostic testing in the presence of predefined indicator diseases across healthcare institutions in Türkiye. The communication included an indicator-disease list, physician and patient information materials, and HIV diagnostic testing algorithms, and stated that testing should be performed after patient information and consent in accordance with confidentiality principles. This official communication represented an important policy development in Türkiye’s HIV response strategy by supporting the integration of indicator-condition–guided testing into routine clinical practice.

Tertiary-level public, university, and training-and-research hospitals serve as regional referral centres with advanced diagnostic capacity and multidisciplinary expertise. These settings provide an epidemiologically informative context for evaluating the distribution of HIV indicator conditions, HIV testing practices, and missed diagnostic opportunities in Türkiye. To ensure broad geographical coverage, tertiary referral hospitals from all seven geographical regions of Türkiye were included. To date, no national multicentre study in Türkiye has systematically quantified the burden of HIV indicator conditions among hospitalised adults or evaluated HIV testing coverage in this population.

Against this background, we conducted a national multicentre point-prevalence survey across tertiary referral hospitals in Türkiye to quantify the prevalence of HIV indicator conditions among hospitalised adults, assess HIV testing coverage when clinically indicated, and identify missed diagnostic opportunities.

## Materials and methods

### Study design and setting

This study was a national multicentre cross-sectional point-prevalence survey conducted in eight tertiary referral hospitals across Türkiye’s seven geographical regions. Tertiary-level public university and training-and-research hospitals were purposively selected to ensure broad geographical coverage and inclusion of centres providing comprehensive HIV diagnostic and inpatient care. One centre was included from each of six geographical regions, and two centres were included from the Black Sea region because of its geographical structure and healthcare access-related considerations. Centre-level characteristics, including geographical region, adult acute-care bed capacity, availability of specialised HIV outpatient care, and infectious diseases physician staffing, are presented in Supplementary Table S1. The study methodology was aligned with the European Centre for Disease Prevention and Control (ECDC) point-prevalence survey protocol (version 5.3)^20^, adapted for an HIV indicator-condition–based assessment framework.

### Preparatory phase

Prior to the main survey, a structured preparatory phase was implemented across all participating centres to ensure methodological consistency across centres. Online training sessions were conducted for site principal investigators and participating physicians to harmonise case definitions, inclusion criteria, indicator-condition classification, and data entry procedures.

A pilot assessment was conducted one week before the index day in selected inpatient units to assess feasibility and inter-observer consistency. Pilot findings were centrally reviewed, and site-specific feedback was provided to optimise procedural consistency prior to full-scale implementation (Supplementary Table S2).

### Study governance and data quality control

Each participating centre designated a senior infectious diseases specialist as site principal investigator, responsible for local coordination and protocol adherence. On the index day, patient identification and indicator-condition assessment were supervised locally. Following data collection, all patient-level entries underwent structured central verification to ensure completeness, internal consistency, and protocol compliance prior to aggregation.

### Study population and index day

The index day was predetermined for all participating centres as 11 February 2026. All adult patients (≥ 18 years) who were already hospitalised and physically present in inpatient wards at the index census time of 08:00 Türkiye time were eligible for inclusion. The 08:00 census time was used as a fixed reference point for single-count enumeration and did not refer to the time of hospital admission or to the exact time of clinical assessment. For patients who met the census-time eligibility criteria, clinical information and HIV testing indications were assessed during the index day and, where necessary, verified from medical records, patient assessment, and the primary clinical team within 24 h of the census time. Patients admitted after the 08:00 census time were excluded. Patients transferred between wards on the same day were evaluated once, according to the ward in which they were present at the 08:00 census time. Day-case procedures (e.g., dialysis, chemotherapy, endoscopy) and patients managed exclusively in emergency department short-stay or observation units were excluded.

The point-prevalence design ensured single-count enumeration and comparability across centres, consistent with established point-prevalence methodology^20^

### HIV testing ındications and ındicator conditions

HIV testing indications were categorised in accordance with European and global HIV testing guidance into three groups: (i) AIDS-defining conditions; (ii) indicator conditions associated with an expected undiagnosed HIV prevalence ≥ 0.1%; and (iii) conditions where unrecognised HIV could substantially affect clinical management (e.g., before immunosuppressive therapy, chemotherapy, or biologic treatment)^14–17^.

AIDS-defining conditions were defined according to the revised CDC surveillance case definition for HIV infection among adolescents and adults^21^. Standardised definitions and inclusion criteria were provided to all participating centres to ensure harmonised classification across sites. The complete list of HIV testing indications used in the study, including AIDS-defining conditions, indicator conditions associated with an expected undiagnosed HIV prevalence ≥ 0.1%, conditions in which unrecognised HIV could substantially affect clinical management, and additional nationally recommended indicator-disease categories, is provided in Supplementary Table S3.

Patients could have more than one HIV testing indication. All documented HIV testing indications were recorded. Descriptive summaries of specific indications were therefore indication-based, and categories were not mutually exclusive. In contrast, patient-level analyses, including HIV testing coverage and analyses of non-performance of HIV testing by the primary clinical team, included each patient only once. For patients with multiple indications, the chronologically first documented HIV testing indication was used as the reference indication in logistic regression and retrospective temporal analyses.

### Data collection

Data were collected using three structured electronic case report forms (eCRFs) developed on a secure web-based platform. Each form was designed to capture a distinct dataset.

Form A (Centre Characteristics Form) recorded hospital-level characteristics, including geographical region, number of adult inpatients on the index day, availability of HIV testing modalities, and local HIV care infrastructure.

Form B (HIV Testing Indication Form) was completed for adult inpatients meeting criteria for at least one predefined HIV testing indication and captured demographic characteristics, admitting department, specific indication(s), whether HIV testing was performed, declined, or not performed (with reasons), and test results. For newly diagnosed individuals, CD4 T-cell count and HIV RNA levels were recorded where available.

Form C (Known HIV-Positive Form) was completed for patients with pre-existing HIV infection and documented the presence of indicator conditions at admission, enabling assessment of prior missed opportunities for HIV testing based on established definitions in the literature^22^.

Adult inpatients present at 08:00 and assessed during the 24-h active-surveillance window constituted the evaluated point-prevalence denominator; detailed patient-level data were collected only for those with ≥ 1 predefined HIV testing indication (Form B) and for those with known HIV infection (Form C).

All eCRFs incorporated mandatory fields and automated logic checks to enhance data completeness and internal consistency.

### Data coding and anonymisation

All patient data were anonymised at the participating centres prior to central aggregation. Unique study identifiers were generated using a structured coding system aligned with national data protection practices to enable case tracking while preventing direct personal identification. No directly identifiable personal data were collected, stored, or transferred to the central database.

### Eligibility criteria

Inclusion criteria were: (i) age ≥ 18 years; and (ii) presence in an inpatient ward at 08:00 on the predetermined index day. All adult inpatients meeting these criteria were considered eligible for active surveillance during the 24-h survey window.

Exclusion criteria were: (i) day-case or outpatient-only encounters; (ii) admissions occurring after the 08:00 census time; (iii) patients managed exclusively in emergency department short-stay or observation units; and (iv) duplicate entries across study forms.

### Study outcomes

The primary outcome was HIV testing coverage among patients with at least one documented indication for HIV testing. Secondary outcomes included: (i) the burden of predefined HIV testing indications in the hospitalised adult cohort; (ii) the frequency and determinants of non-performance of HIV testing among patients with a documented indication; (iii) prior missed diagnostic opportunities among hospitalised people living with HIV; and (iv) HIV test positivity, including the proportion of previously undiagnosed infections. A descriptive retrospective temporal assessment was performed as an exploratory contextual analysis.

### Data management and quality control

All data were stored on secure institutional servers with restricted access limited to authorised study personnel. Unique anonymised identifiers were used to maintain confidentiality during central aggregation. Data completeness and consistency were monitored centrally.

Quality assurance measures included pre-study training of all research teams, provision of standardised indicator-condition definitions, automated form validation within the electronic data capture system, and a random 5% cross-check audit to assess inter-rater reliability and data accuracy. Any discrepancies identified during verification were resolved through central adjudication in consultation with the relevant site principal investigator.

### Ethics approval and consent to participate

The study was approved by the Istanbul Medipol University Non-Interventional Clinical Research Ethics Committee before patient inclusion and data collection (Decision No: 1214; 21 October 2025). Local institutional permissions and, where required, local approvals from participating centres were obtained before the index day. Patient inclusion and data collection were conducted on the predetermined index day, 11 February 2026.

All data were fully anonymised prior to analysis, and no directly identifiable information was recorded or transferred. HIV testing was conducted in accordance with routine national clinical care protocols. No patient included in the evaluated cohort refused consent for study participation or data assessment. Patients who declined HIV testing after patient information were retained in the evaluated cohort for clinical, indication-based, and risk-factor assessments, but were classified as not tested in the HIV testing cascade; the clinical characteristics and reasons for residual non-testing are reported in Supplementary Table S4.

The study was performed in accordance with the principles of the Declaration of Helsinki.

### Statistical analysis

Continuous variables are reported as medians and interquartile ranges (IQRs), whereas categorical variables are reported as counts and percentages.

HIV testing coverage among patients with documented indication for HIV testing was estimated with corresponding 95% confidence intervals (CIs). Between-group comparisons were performed using χ^2^ or Fisher’s exact tests for categorical variables.

To identify factors associated with non-performance of HIV testing by the primary clinical team, univariable and multivariable logistic regression analyses were performed. The binary outcome was defined as non-performance of HIV testing by the primary clinical team despite documented indication for HIV testing. Results are reported as odds ratios (ORs) and adjusted odds ratios (aORs) with 95% CIs.

Temporal patterns in HIV testing performance were assessed using a descriptive retrospective temporal analysis among patients with documented HIV testing indications identified in the point-prevalence cohort. This analysis was not based on repeated point-prevalence surveys. Instead, after obtaining patient consent, previous hospital records, clinical history, HIV test-order information, and available data from the national electronic health record system in Türkiye were reviewed to determine whether HIV testing had been requested when a relevant testing indication was present. Calendar-time patterns in testing performance were then described according to the timing of documented testing indications and HIV test requests. Trends were visualised using locally weighted regression (LOESS) smoothing curves to illustrate overall temporal patterns. The COVID-19 disruption period (1 March 2020–1 January 2023) and the official institutional communication issued by the Republic of Türkiye Ministry of Health, General Directorate of Public Health, on 30 June 2025 were prespecified as contextual reference points.

All statistical tests were two-sided, and p < 0.05 was considered statistically significant. Analyses were performed using R (version 4.5.3)^23^. The study is reported in accordance with the STROBE statement^24^, and the completed STROBE checklist is provided as Supplementary File S1.

## Results

### Study population and testing cascade

**Fig. 1:**
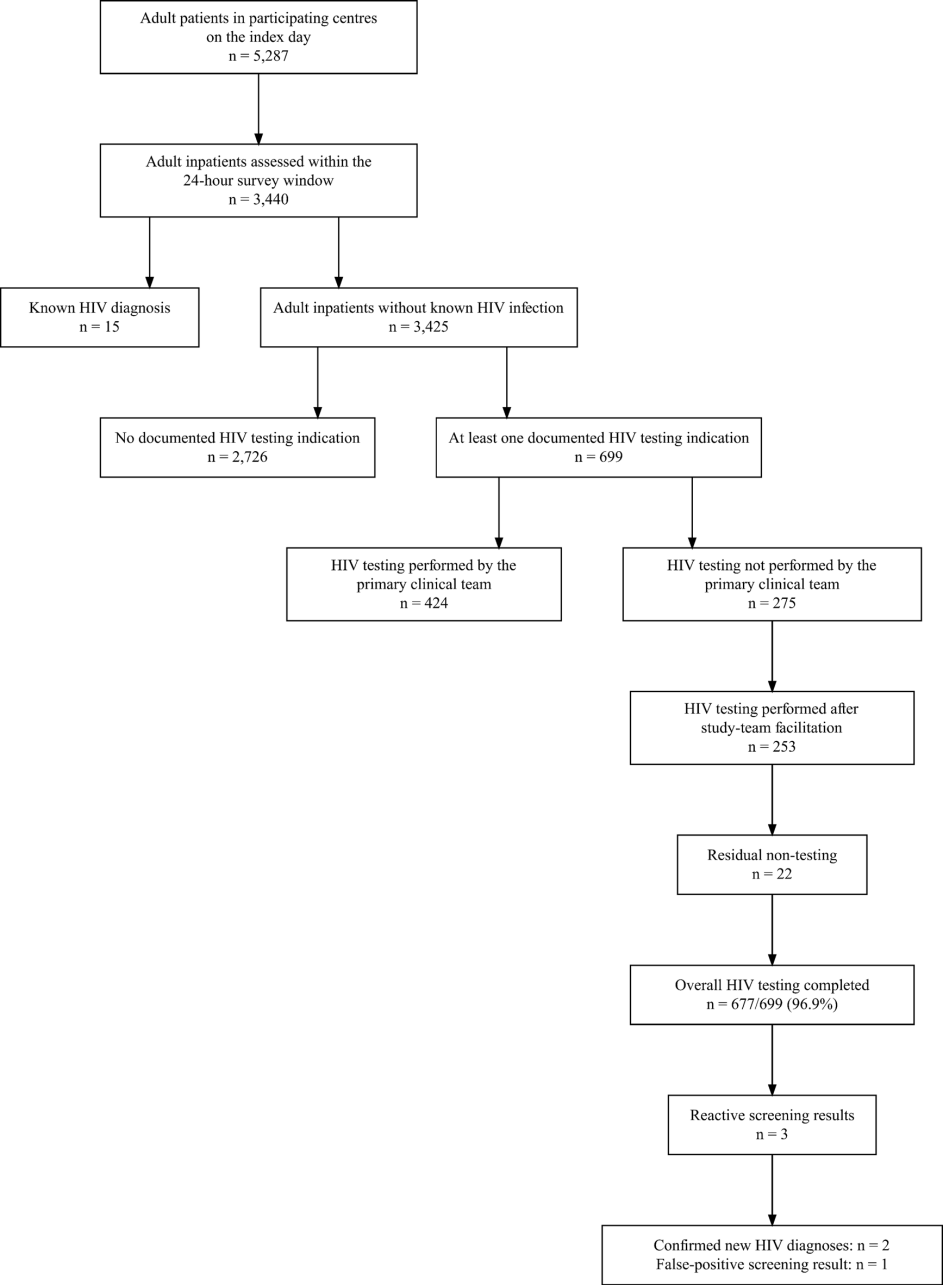
Study flow diagram and HIV testing cascade. The flow diagram shows the adult inpatient cohort assessed during the 24-h survey window, exclusion of patients with known HIV infection, identification of patients with documented HIV testing indications, HIV testing performed by the primary clinical team, additional testing after study-team facilitation, residual non-testing, and final HIV screening outcomes. Abbreviations: HIV, human immunodeficiency virus.

On the predefined index day, 5,287 adult patients were hospitalised across participating centres. Within the 24-h survey window, 3,440 adult inpatients who were present in eligible inpatient wards at the 08:00 census time could be assessed through active surveillance and constituted the evaluated cohort, including 15 individuals with a known HIV diagnosis. These 15 individuals were excluded from analyses evaluating testing indications and testing performance. Among the remaining patients (n = 3,425), 699 met criteria for at least one documented testing indication. HIV testing was performed by the primary clinical team in 424 of these eligible patients (60.7%) prior to study-team involvement. Of the 275 patients who had not undergone testing despite documented indications, 253 underwent HIV testing during the point-prevalence survey, while testing remained unperformed in 22 patients (3.1%). Overall, three reactive screening test results were identified, including two confirmed new HIV diagnoses and one false-positive result (Fig. 1).

### People living with HIV and prior missed diagnostic opportunities

A total of 15 hospitalised people living with HIV (PLWH) were identified among the assessed cohort on the index day. Eleven were male and four were female, aged 21–64 years. CD4 T-cell counts at HIV diagnosis ranged from 10 to 550 cells/mm^3^.

Six PLWH had documented AIDS-defining or other recognised HIV testing indications before HIV diagnosis, representing potential missed or delayed diagnostic opportunities, including cryptococcal meningitis, Pneumocystis jirovecii pneumonia, pulmonary or genitourinary tuberculosis, herpes zoster, and unexplained cognitive impairment. HIV testing had been performed at initial presentation in four of these cases (cryptococcal meningitis, Pneumocystis jirovecii pneumonia, and tuberculosis), with intervals of 1–2 months between presentation and diagnosis. In contrast, HIV testing was not performed in two individuals presenting with herpes zoster and unexplained cognitive impairment, resulting in diagnostic delays of 7 and 11 months, respectively. Individual-level characteristics of hospitalised people living with HIV are provided in Supplementary Table S5.

### Characteristics of patients with HIV testing ındications and primary clinical team testing

Among patients with documented indication for HIV testing (n = 699), the median age was 60 years (IQR, 45–70), and 372 (53.2%) were male. A specialised HIV outpatient clinic was available in 426 cases (60.9%). Self-reported HIV risk factors were documented in 32 patients (4.6%), most commonly blood transfusion before routine HIV screening (10 [31.2%]), multiple sexual partners (6 [18.8%]), and sexually transmitted infections (6 [18.8%]). A total of 568 patients (81.3%) reported previous HIV testing, with a median of 2 prior negative tests (IQR, 1–5).

Within this cohort, 90 patients (12.9%) had AIDS-defining conditions (ADC), 278 (39.8%) had conditions in which unrecognised HIV could substantially affect clinical management, and 289 (41.3%) had indicator conditions with an expected undiagnosed HIV prevalence ≥ 0.1%. Pregnancy was identified as an additional indication for HIV testing in 44 patients.

The most common AIDS-defining conditions were non-Hodgkin lymphoma (42 [45.7%]), progressive multifocal leukoencephalopathy (15 [16.3%]), and invasive cervical cancer (11 [12.0%]). Among conditions in which unrecognised HIV could substantially affect clinical management, solid malignancies receiving chemotherapy were the most common (211 [75.9%]), followed by autoimmune diseases treated with immunosuppressive therapy (33 [11.9%]) and hematologic malignancies receiving systemic chemotherapy (20 [7.2%]). Among indicator conditions with an expected undiagnosed HIV prevalence ≥ 0.1%, severe bacterial pneumonia (112 [37.8%]), hepatitis B infection (46 [15.5%]), and herpes zoster (22 [7.4%]) were most common.

Overall, HIV testing was performed by the primary clinical team in 424 of 699 patients with documented testing indications (60.7%). By indication category, testing was performed in 67 of 90 patients with AIDS-defining conditions (74.4%), 176 of 278 patients with conditions in which unrecognised HIV could substantially affect clinical management (63.3%), and 138 of 289 patients with indicator conditions associated with an expected undiagnosed HIV prevalence ≥ 0.1% (47.8%) (Table 1).

**Table 1:** Characteristics of hospitalised adults and HIV testing performed by the primary clinical team prior to the point-prevalence assessment.

Characteristic	Hospitalised adults without known HIV infection	HIV testing indicated (≥ 1 indication)	Primary clinical team–performed HIV testing
Hospitalised adults without known HIV infection evaluated within the 24-h survey window, n	3425	699	424
Age, median [Q1–Q3]	60.0 [44.0–70.0]	60.0 [45.0–70.0]	58.0 [40.0–69.0]
Female sex, n (%)	1664 (48.6%)	327 (46.8%)	219 (51.9%)
Specialised HIV outpatient clinic available, n (%)	1420 (41.5%)	426 (60.9%)	253 (60.0%)
Self-reported HIV risk factors present, n (%)	32 (0.9%)	32 (4.6%)	20 (4.7%)
Blood transfusion before routine HIV screening	10 (31.2%)	10 (31.2%)	7 (35.0%)
Multiple sexual partners	6 (18.8%)	6 (18.8%)	3 (15.0%)
Sexually transmitted infections	6 (18.8%)	6 (18.8%)	4 (20.0%)
History of incarceration	5 (15.6%)	5 (15.6%)	3 (15.0%)
People who inject drugs (PWID)	2 (6.2%)	2 (6.2%)	1 (5.0%)
Other^a^	3 (9.4%)	3 (9.4%)	2 (10.0%)
Self-reported previous HIV testing, n (%)	1248 (36.4%)	568 (81.3%)	390 (92.4%)
Number of documented previous negative HIV tests among patients reporting previous HIV testing, median [Q1–Q3]	2.0 [1.0–5.0]	2.0 [1.0–5.0]	2.0 [1.0–5.0]
AIDS-defining conditions, n (%)	90 (2.6%)	90 (12.9%)	67 (15.9%)
Non-Hodgkin lymphoma	42 (45.7%)	42 (45.7%)	36 (52.2%)
Progressive multifocal leukoencephalopathy	15 (16.3%)	15 (16.3%)	12 (17.4%)
Invasive cervical cancer	11 (12.0%)	11 (12.0%)	10 (14.5%)
Tuberculosis	10 (10.9%)	10 (10.9%)	6 (8.7%)
Esophageal candidiasis	4 (4.3%)	4 (4.3%)	0 (0.0%)
Other^b^	10 (10.9%)	10 (10.9%)	5 (7.2%)
Conditions in which unrecognised HIV could substantially affect clinical management, n (%)	278 (8.1%)	278 (39.8%)	176 (41.7%)
Solid malignancies receiving chemotherapy	211 (75.9%)	211 (75.9%)	131 (74.4%)
Autoimmune disease treated with immunosuppressive therapy	33 (11.9%)	33 (11.9%)	13 (7.4%)
Hematologic malignancy receiving systemic chemotherapy	20 (7.2%)	20 (7.2%)	18 (10.2%)
Solid organ transplant recipients	10 (3.6%)	10 (3.6%)	10 (5.7%)
Hematopoietic stem cell transplantation (HSCT)	4 (1.4%)	4 (1.4%)	4 (2.3%)
Indicator conditions with expected undiagnosed HIV prevalence ≥ 0.1% present, n (%)	289 (8.4%)	289 (41.3%)	138 (32.7%)
Severe bacterial pneumonia	112 (37.8%)	112 (37.8%)	36 (25.0%)
Hepatitis B infection	46 (15.5%)	46 (15.5%)	29 (20.1%)
Herpes zoster	22 (7.4%)	22 (7.4%)	10 (6.9%)
Unexplained weight loss	12 (4.1%)	12 (4.1%)	8 (5.6%)
Unexplained cytopenia (leukopenia or thrombocytopenia ≥ 4 weeks)	11 (3.7%)	11 (3.7%)	4 (2.8%)
Other^c^	93 (31.4%)	93 (31.4%)	57 (39.6%)
Pregnancy, n (%)	44 (1.3%)	44 (6.3%)	42 (10.0%)
Admitting department, n (%)			
Internal medicine departments	1504 (43.9%)	422 (60.4%)	254 (60.2%)
Surgical departments	1400 (40.9%)	162 (23.2%)	88 (20.9%)
Adult Intensive Care Unit	296 (8.6%)	65 (9.3%)	35 (8.3%)
Obstetrics and Gynecology	148 (4.3%)	44 (6.3%)	41 (9.7%)
Psychiatry	77 (2.2%)	6 (0.9%)	4 (0.9%)

Self-reported HIV risk factors were calculated among patients reporting at least one HIV risk factor. Percentages for the “HIV testing indicated” column were calculated among patients with documented testing indications (n = 699), and percentages for the “Primary clinical team–performed HIV testing” column were calculated using the corresponding column total (n = 424) as the denominator. All patients reporting self-identified HIV exposure risk were included in the subgroup with documented HIV testing indications; therefore, subtype counts and percentages are identical in the overall cohort and the indicated subgroup. Indication categories and subcategories were not mutually exclusive; therefore, totals may exceed the number of patients with documented indications. Percentages for subcategories of indication groups were calculated using the total number of documented indications within the corresponding indication group as the denominator.

^a^Other self-reported HIV risk factors included being born in or having lived in a high HIV-prevalence country and unprotected sexual intercourse in the past 6 months.

^b^Other AIDS-defining conditions included cerebral toxoplasmosis, Pneumocystis jirovecii pneumonia, cryptococcal meningitis, cytomegalovirus retinitis, chronic herpes simplex virus ulceration (> 1 month), candidiasis of bronchi, trachea, or lungs, and disseminated histoplasmosis.

^c^Other indicator conditions included sexually transmitted infection, candidemia, peripheral neuropathy, hepatitis C infection, fever of unknown origin, Guillain–Barré syndrome, invasive pneumococcal disease, oropharyngeal candidiasis, high-grade anal dysplasia/anal cancer, severe or atypical psoriasis, severe seborrheic dermatitis, unexplained mononeuritis multiplex, subcortical dementia, and unexplained chronic diarrhoea.

The number of documented previous negative HIV tests was calculated among patients reporting previous HIV testing; patients without reported previous HIV testing were not assigned a value of zero for this summary.

ADC, AIDS-defining condition; HIV, human immunodeficiency virus; HSCT, hematopoietic stem cell transplantation; IC, indicator condition; ICU, intensive care unit; IQR, interquartile range; PWID, people who inject drugs.

### Factors associated with non-performance of HIV testing by the primary clinical team

In univariable analyses, increasing age was associated with higher odds of non-performance of HIV testing by the primary clinical team (OR 1.02 per 1-year increase, 95% CI 1.01–1.03; p < 0.001), as was male sex (OR 1.67, 95% CI 1.23–2.28; p = 0.001). By contrast, a history of previous HIV testing was associated with lower odds of non-performance (OR 0.12, 95% CI 0.07–0.22; p < 0.001). Compared with AIDS-defining conditions (ADC), indicator conditions associated with an expected undiagnosed HIV prevalence ≥ 0.1% were likewise associated with higher odds of non-performance of HIV testing by the primary clinical team (OR 3.19, 95% CI 1.91–5.50; p < 0.001).

**Fig. 2:**
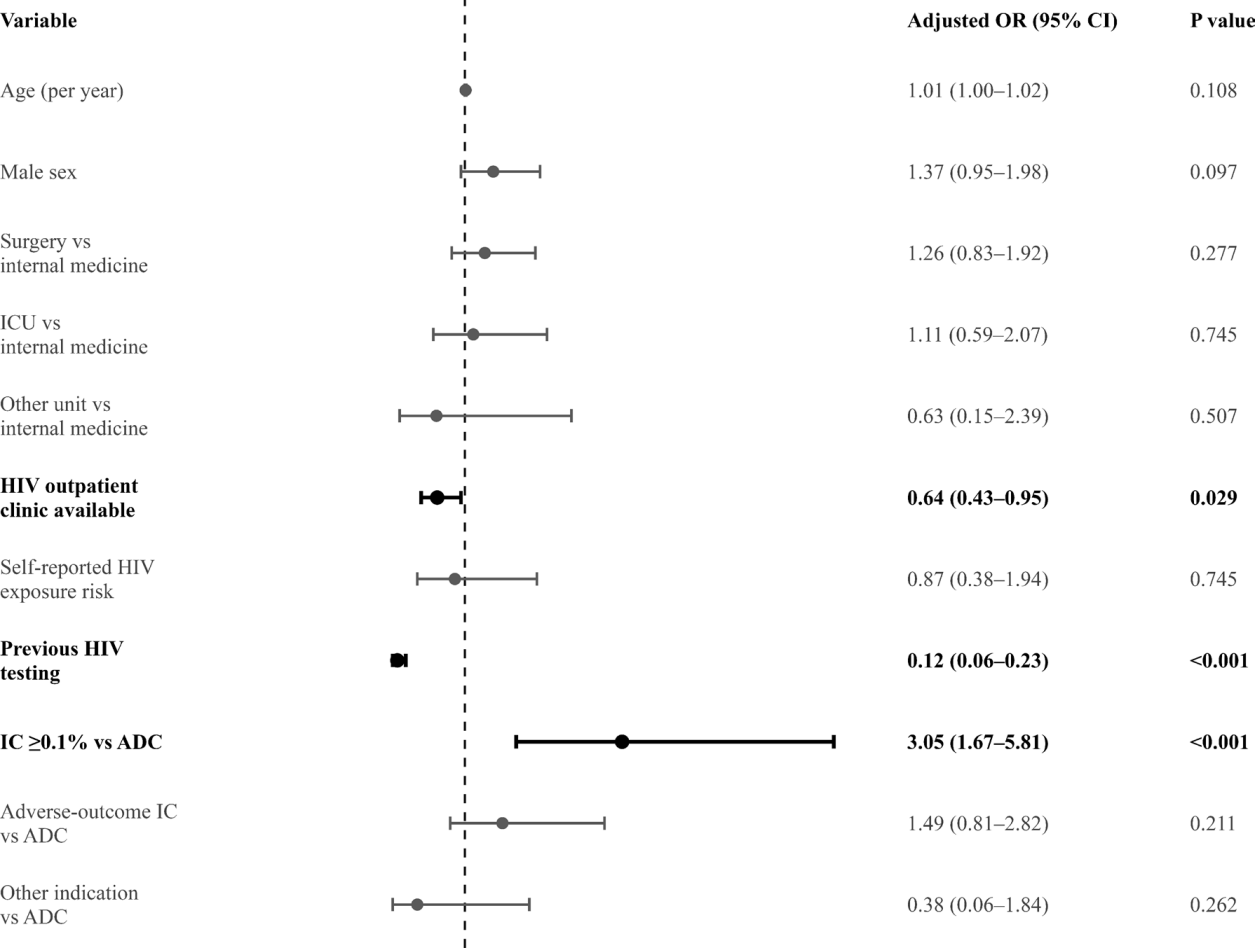
Factors associated with non-performance of HIV testing by the primary clinical team. Forest plot showing adjusted odds ratios, 95% confidence intervals, and P values from the multivariable logistic regression model. The outcome was non-performance of HIV testing by the primary clinical team despite a documented indication for HIV testing. The analysis was restricted to patients with documented HIV testing indications. The vertical dashed line indicates an aOR of 1. Estimates to the right of the line indicate higher odds of non-performance, whereas estimates to the left indicate lower odds of non-performance. Bold text and darker plot elements indicate associations with 95% confidence intervals not crossing 1. For patients with multiple HIV testing indications, the chronologically first documented indication was used as the reference indication for patient-level regression analysis. Abbreviations: ADC, AIDS-defining condition; aOR, adjusted odds ratio; CI, confidence interval; IC, indicator condition; ICU, intensive care unit.

In multivariable analysis, previous HIV testing remained independently associated with lower odds of non-performance of HIV testing by the primary clinical team (aOR 0.12, 95% CI 0.06–0.23; p < 0.001). By contrast, compared with AIDS-defining conditions (ADC), indicator conditions associated with an expected undiagnosed HIV prevalence ≥ 0.1% remained independently associated with higher odds of non-performance (aOR 3.05, 95% CI 1.67–5.81; p < 0.001). Availability of a specialised HIV outpatient clinic was also independently associated with lower odds of non-performance (aOR 0.64, 95% CI 0.43–0.95; p = 0.029). Age and sex were not independently associated with non-performance after adjustment. The adjusted associations are summarised visually in Fig. 2.

### Residual missed HIV testing after study-team ınvolvement

Among the 275 patients with documented HIV testing indications who had not undergone HIV testing by the primary clinical team, testing was performed in 253 individuals during the point-prevalence survey. In 22 patients, HIV testing could not be performed despite documented testing indications.

The most frequent documented indication for HIV testing among these patients was severe bacterial pneumonia (n = 9), followed by autoimmune disease receiving immunosuppressive therapy (n = 2) and hepatitis B virus infection (n = 2).

The primary reason for residual non-testing was refusal of HIV testing after patient information (n = 16), most commonly due to imminent discharge (n = 10) or self-perceived low risk (n = 6); individual-level clinical characteristics and reasons for residual missed HIV testing are provided in Supplementary Table S4.

In six cases, HIV testing was not completed due to clinical or organisational factors, including cases in which testing was not recommended during study-team review (n = 5) or was declined by the primary clinical team (n = 1).

Overall, HIV testing was completed in 677 of 699 patients with documented testing indications (96.9%), whereas 22 patients (3.1%) remained untested.

### Descriptive retrospective temporal assessment of HIV testing performance

A descriptive retrospective temporal assessment was performed using previous clinical history, hospital records, HIV test-order information, and available national electronic health record data among patients with documented HIV testing indications in the point-prevalence cohort. This analysis did not represent national HIV testing trends and was not based on repeated point-prevalence surveys. Rather, it was intended to provide contextual information on whether clinically indicated HIV testing had been requested over calendar time among patients included in the present study. Weekly testing proportions showed temporal variation across calendar years, with lower observed values during the early pandemic-related disruption period and a subsequent pattern of recovery. Observed testing performance was generally higher in the later calendar years, particularly from late 2023 onward. Because this analysis was descriptive and retrospective, it should not be interpreted as establishing a causal effect of the national guidance issued on 30 June 2025. The corresponding figure has been moved to the Supplementary Information as Supplementary Figure S1.

## Discussion

This national multicentre point-prevalence survey across tertiary referral hospitals in Türkiye identified a substantial implementation gap in clinically indicated HIV testing among hospitalised adults. In routine care, HIV testing was requested by the primary clinical team for 60.7% of patients with documented indications for testing, whereas testing uptake increased to 96.9% when testing was actively prompted and facilitated through the survey process. These findings suggest that the main challenge in inpatient settings is not only recognising HIV testing indications, but ensuring that these indications are translated into timely test requests and completed testing within routine clinical workflows.

In a multicentre European hospital implementation study, baseline testing rates were approximately 40%–50% and increased during the intervention period from 40.2 to 49.4%, indicating that implementation support can improve testing uptake, although often incrementally^25^. Against this background, the routine inpatient testing rate observed in the present survey, with HIV testing requested by the primary clinical team in 60.7% of patients with documented indications for HIV testing, falls within the broader range of published hospital experience but still indicates substantial room for improvement. By contrast, when testing was actively prompted and facilitated through the survey process, including recommendations from infectious diseases specialists, overall testing uptake increased markedly to 96.9%.

Missed opportunities for HIV testing are clinically important because they can delay diagnosis even in patients who have already engaged with healthcare services, prolonging the time to treatment initiation and increasing the likelihood of advanced presentation at diagnosis^22^. In this survey, a similar pattern was observed among hospitalised people living with HIV identified on the index day: several had documented AIDS-defining conditions or other recognised HIV testing indications before HIV diagnosis, and although HIV testing had been requested at the initial presentation in some cases, it had not been requested in others despite the presence of a recognised indication, including patients presenting with herpes zoster and unexplained cognitive impairment. Although based on small numbers, these findings provide a clinically meaningful illustration of how missed testing opportunities can persist in hospital care and contribute to delayed HIV diagnosis.

Across European hospital-based studies, HIV testing uptake among patients with indicator conditions has remained suboptimal, with reported testing rates of approximately 40%–50% in routine or baseline practice and a median testing rate of 72% across audited centres^17, 18, 25^. In the present survey, the primary clinical team requested HIV testing in 47.8% of patients with indicator conditions associated with an expected undiagnosed HIV prevalence ≥ 0.1%, and this was the lowest testing-request rate among the predefined indication categories. This finding is clinically important because these conditions are central to contemporary indicator-condition–guided testing strategies^14–17^, yet they may be less likely than AIDS-defining presentations to trigger an immediate HIV testing reflex in routine inpatient practice. The findings therefore support a more implementation-focused approach, in which indicator-condition lists are embedded into routine workflows through consistent prompting and test-request pathways, rather than relying on clinician recognition alone. In Türkiye’s non-generalised HIV epidemic context, indicator-condition–guided testing therefore remains a pragmatic targeted strategy. A universal inpatient testing strategy might identify additional infections among patients without recognised or documented indicator conditions; however, the expected positivity per test would probably be lower than in a clinically targeted approach, and its additional yield, feasibility, acceptability, laboratory capacity requirements, and cost-effectiveness would require formal evaluation.

Evidence from European hospital-based studies indicates that HIV testing performance when clinically indicated is shaped not only by the presenting condition but also by testing practices and institutional capacity^18, 25^. In the present survey, patients with a history of previous HIV testing were less likely to experience non-performance of HIV testing by the primary clinical team, while indicator conditions associated with an expected undiagnosed HIV prevalence ≥ 0.1% remained linked to higher odds of non-performance compared with AIDS-defining conditions. Availability of a specialised HIV outpatient clinic was also associated with lower odds of non-performance. These findings suggest that both prior engagement with testing and institutional readiness, such as the presence of clear referral and follow-up pathways, may facilitate the translation of documented HIV testing indications into routine clinical action. Together, these findings indicate that reducing non-performance requires not only clinician-level prompting but also system-level support embedded within routine inpatient care pathways. This may help explain the small residual testing gap observed even after active facilitation during the survey process.

Previous studies examining missed opportunities for HIV diagnosis have shown that, even when indicator condition guided testing strategies, educational interventions, or hospital-based implementation programmes are introduced, a proportion of clinically indicated HIV tests may still remain unperformed because of patient level or operational barriers within routine clinical care^17, 18, 22, 25^. In the present survey, a similarly small residual gap persisted despite active facilitation during the study process, with only a small proportion of patients with documented HIV testing indications not undergoing testing. The reasons included patient refusal, patients perceiving themselves as belonging to a low risk group, imminent discharge, and occasional testing declined by the primary clinical team. Although numerically limited, these observations suggest that improving HIV testing coverage requires attention not only to clinician awareness and institutional readiness but also to practical barriers that may arise during routine inpatient care. Addressing such barriers through streamlined testing pathways and timely test ordering may help further reduce residual missed opportunities for HIV diagnosis in hospital settings.

Evidence from European hospital-based studies indicates that structured implementation strategies, including clinician education and indicator-condition–guided testing programmes, can improve HIV testing uptake when supported by institutional pathways and clinician prompting^2, 18, 25^. In the PROTEST programme, testing uptake increased from approximately 46% at baseline to about 63% following implementation measures in hospital settings^25^. In the present survey, testing increased markedly when infectious diseases specialists actively facilitated testing during the survey process, with the proportion of patients with documented testing indications who ultimately underwent HIV testing exceeding 96%. The descriptive retrospective temporal assessment provides contextual information on testing performance over calendar time but should not be interpreted as evidence of a causal effect of national guidance. Together, these findings highlight the importance of structured clinical pathways and specialist-supported implementation in translating documented HIV testing indications into completed testing within routine hospital care.

This study has several important strengths. It represents a national multicentre assessment conducted across tertiary referral hospitals from all seven geographical regions of Türkiye, providing broad geographical representation and enabling evaluation of HIV testing practices across diverse hospital settings. The study applied a standardised indicator-condition–based framework aligned with international HIV testing guidance, supported by pre-study training, pilot assessment, and structured electronic data collection with central verification procedures, which enhanced methodological consistency and data quality across participating centres. In addition, the study design enabled a comprehensive evaluation of HIV testing practices across the hospital testing cascade by integrating assessment of testing indications, routine testing performance, missed diagnostic opportunities, and residual barriers to testing within the same inpatient population. The inclusion of specialist-facilitated testing during the survey process also allowed comparison between routine clinical practice and facilitated testing uptake, providing implementation-relevant insight into how clinical support and institutional pathways may improve testing coverage. Finally, the combination of a national point-prevalence survey with a descriptive retrospective temporal assessment provides additional contextual information on clinically indicated HIV testing performance among patients included in the study.

This study has several limitations. First, the point-prevalence design captures HIV testing indications and testing practices at a single time point and therefore does not allow assessment of longitudinal clinical trajectories or causal relationships. Second, because active surveillance was conducted within a predefined 24-h survey window, the findings reflect the actively evaluated patient cohort. Third, participating centres were tertiary referral hospitals with advanced diagnostic capacity, and therefore the findings may not fully reflect HIV testing practices in secondary-level or smaller healthcare facilities. Fourth, the descriptive retrospective temporal assessment was exploratory and based on historical clinical and electronic health record information among patients included in the point-prevalence cohort. It should not be interpreted as representing national HIV testing trends or establishing causal effects of national guidance. Fifth, the study was not designed to directly compare indicator-condition–guided testing with universal inpatient HIV testing; therefore, the findings cannot determine the additional yield, cost-effectiveness, or acceptability of universal testing in routine inpatient workflows in Türkiye. Finally, information on HIV exposure risk factors relied partly on self-reported data documented in medical records, which may be subject to underreporting or incomplete documentation. Despite these limitations, the study provides a comprehensive national overview of indicator-condition–guided HIV testing practices in hospitalised adults and highlights clinically relevant gaps in testing implementation within routine inpatient care.

## Conclusion

This national multicentre point-prevalence survey demonstrates that HIV testing indications are common among hospitalised adults in Türkiye, yet these indications are not consistently translated into routine clinical testing. In particular, indicator conditions associated with an expected undiagnosed HIV prevalence ≥ 0.1% were associated with higher odds of non-performance of HIV testing by the primary clinical team, revealing an important implementation gap in indicator-condition–guided testing strategies. The marked improvement observed when testing was actively facilitated by infectious diseases specialists suggests that specialist-supported implementation may help translate documented testing indications into completed HIV testing. The descriptive retrospective temporal assessment provides contextual information on testing performance over calendar time but should not be interpreted as evidence of a causal effect of national guidance. Integrating indicator-condition prompts into routine clinical workflows and reinforcing institutional testing pathways may help reduce missed diagnostic opportunities and promote earlier HIV diagnosis among hospitalised adults.

## Supplementary Information

Supplementary Information.

Supplementary Information.

Supplementary Information.

Supplementary Information.

Supplementary Information.

## Data Availability

The datasets generated during and/or analysed during the current study are not publicly available due to institutional and participant confidentiality considerations but are available from the corresponding author on reasonable request.
