# Supplementary material for: Missed Opportunities for HIV Testing in Hospitalised Adults in Türkiye: Indicator Conditions and Testing Coverage in a National Multicentre Point-Prevalence Survey (HIV-ICs-TR)
Source: Sci Rep. 2026 May 30;16:23715. doi: 10.1038/s41598-026-54294-6 (PMC13427839; doi:10.1038/s41598-026-54294-6)
Supplement: Supplementary file 1 — Supplementary Information. [file 41598_2026_54294_MOESM1_ESM.docx]

**Supplementary Table S1** Characteristics of participating centres in the HIV-ICs-TR point-prevalence survey

| **Centre** | **Geographic region** | **Specialised HIV outpatient clinic available** | **Adult acute-care beds, n** | **Infectious diseases staffing, n** |
| --- | --- | --- | --- | --- |
| Centre 1 | Central Anatolia Region | No | 960 | 2 professors, 1 associate professor, 1 assistant professor, 8 residents |
| Centre 2 | Black Sea Region | Yes | 831 | 2 prof., 2 assist. prof., 8 residents |
| Centre 3 | Southeastern Anatolia Region | No | 861 | 3 prof., 1 assoc. prof., 1 assist. prof., 16 residents |
| Centre 4 | Marmara Region | Yes | 590 | 3 prof., 2 assoc. prof., 22 residents |
| Centre 5 | Eastern Anatolia Region | No | 1,100 | 3 professors, 2 associate professors, 24 residents |
| Centre 6 | Aegean Region | Yes | 1,600 | 6 prof., 1 assoc. prof., 1 assist. prof., 19 residents |
| Centre 7 | Mediterranean Region | Yes | 494 | 4 prof., 2 assist. prof., 18 residents |
| Centre 8 | Black Sea Region | No | 470 | 1 prof., 2 assoc. prof., 1 assist. prof., 2 specialists, 10 residents |
| **Total** | **—** | **4 of 8 centres** | **6,906** | **—** |
| Note. Data are presented at the centre level. Participating centres were anonymised as Centre 1–Centre 8. Geographic regions are reported according to the conventional seven geographical regions of Türkiye. Adult acute-care beds refer to adult inpatient acute-care capacity and exclude day-case units, emergency observation areas, operating/recovery rooms, and long-term care, palliative care, and rehabilitation units. Infectious diseases staffing was reported by each participating centre. | | | | |
| Abbreviations: Assoc. Prof., associate professor; Assist. Prof., assistant professor; HIV, human immunodeficiency virus; Prof., professor. | | | | |
