# Supplementary material for: Missed Opportunities for HIV Testing in Hospitalised Adults in Türkiye: Indicator Conditions and Testing Coverage in a National Multicentre Point-Prevalence Survey (HIV-ICs-TR)
Source: Sci Rep. 2026 May 30;16:23715. doi: 10.1038/s41598-026-54294-6 (PMC13427839; doi:10.1038/s41598-026-54294-6)
Supplement: Supplementary file 2 — Supplementary Information. [file 41598_2026_54294_MOESM2_ESM.docx]

**Supplementary Table S2** Pilot point-prevalence cohort: HIV testing indications, testing pathway and outcomes

| **Variable** | **N = 78** |
| --- | --- |
| Age, years | 54 [24–94] |
| Sex |  |
| Male | 40 (51.28%) |
| Self-reported HIV risk factors |  |
| Blood/blood component transfusion in high-risk periods/countries | 1 (1.28%) |
| Multiple sexual partners / high partner turnover | 2 (2.56%) |
| Previous HIV testing | 22 (28.21%) |
| Number of previous negative HIV tests | 1.00 [0.00–9.00] |
| Number of prior healthcare visits for the indicator condition | 1.5 [1.0–20.0] |
| Time since onset of indicator condition to assessment, days | 53 [2–72] |
| Condition requiring aggressive immunosuppressive therapy  Cancer  Autoimmune disease–related immunosuppression  Transplantation | 12 (15.38%)  7 (8.97%)  4 (5.13%)  1 (1.28%) |
| Indicator condition(s) present in the cohort  Herpes zoster  Invasive pneumococcal disease  Non-Hodgkin lymphoma  HBV infection  Severe psoriasis  Tuberculosis  Chronic mucocutaneous HSV ulcer (≥1 month)  Mononeuritis multiplex  Mycosis fungoides  Peripheral neuropathy  Seborrheic dermatitis  Syphilis | 24 (30.77%)  6 (7.69%)  3 (3.85%)  3 (3.85%)  2 (2.56%)  2 (2.56%)  2 (2.56%)  1 (1.28%)  1 (1.28%)  1 (1.28%)  1 (1.28%)  1 (1.28%)  1 (1.28%) |
| HIV testing requested by the primary clinical team  HIV testing recommended by the study team | 26 (33.33%)  10 (12.82%) |
| HIV test refusal despite indication | 4 (5.13%) |
|  |  |

**Note:** Values are presented as median [range] or n (%). Percentages were calculated using the full pilot cohort as the denominator (N = 78), unless otherwise specified. Indicator conditions were not mutually exclusive; therefore, totals may exceed the number of patients in the pilot cohort. The number of previous negative HIV tests was calculated among patients reporting previous HIV testing. All HIV test refusals were due to self-perceived low HIV risk and refusal of repeat blood sampling.

**Abbreviations:** HBV, hepatitis B virus; HIV, human immunodeficiency virus; HSV, herpes simplex virus.
