# Supplementary material for: Missed Opportunities for HIV Testing in Hospitalised Adults in Türkiye: Indicator Conditions and Testing Coverage in a National Multicentre Point-Prevalence Survey (HIV-ICs-TR)
Source: Sci Rep. 2026 May 30;16:23715. doi: 10.1038/s41598-026-54294-6 (PMC13427839; doi:10.1038/s41598-026-54294-6)
Supplement: Supplementary file 3 — Supplementary Information. [file 41598_2026_54294_MOESM3_ESM.docx]

# Supplementary Table S3. Complete list of HIV testing indications used in the HIV-ICs-TR point-prevalence survey

| **Study framework category** | **Indicator-condition group** | **Specific HIV testing indication** |
| --- | --- | --- |
| AIDS-defining conditions | Neoplasms | Kaposi sarcoma |
| AIDS-defining conditions | Neoplasms | AIDS-related non-Hodgkin lymphoma |
| AIDS-defining conditions | Neoplasms | Invasive cervical cancer |
| Indicator conditions with expected undiagnosed HIV prevalence ≥0.1% | Neoplasms | Cervical cancer or cervical dysplasia |
| Indicator conditions with expected undiagnosed HIV prevalence ≥0.1% | Neoplasms | Anal cancer or anal dysplasia |
| Indicator conditions with expected undiagnosed HIV prevalence ≥0.1% | Neoplasms | Primary lung cancer |
| Conditions in which unrecognised HIV could affect clinical management | Before or during major immunosuppression | Solid malignancy receiving systemic chemotherapy |
| Conditions in which unrecognised HIV could affect clinical management | Before or during major immunosuppression | Hematologic malignancy receiving systemic chemotherapy |
| Conditions in which unrecognised HIV could affect clinical management | Before or during major immunosuppression | Solid organ transplantation |
| Conditions in which unrecognised HIV could affect clinical management | Before or during major immunosuppression | Hematopoietic stem cell transplantation |
| Conditions in which unrecognised HIV could affect clinical management | Before or during major immunosuppression | Autoimmune disease requiring immunosuppressive therapy |
| AIDS-defining conditions | Viral infections | Cytomegalovirus retinitis |
| AIDS-defining conditions | Viral infections | Cytomegalovirus disease other than liver, spleen, or lymph nodes |
| AIDS-defining conditions | Viral infections | Herpes simplex ulcer lasting >1 month, bronchitis, pneumonitis, or oesophagitis |
| AIDS-defining conditions | Viral infections | Progressive multifocal leukoencephalopathy |
| Indicator conditions with expected undiagnosed HIV prevalence ≥0.1% | Viral infections | Herpes zoster |
| Indicator conditions with expected undiagnosed HIV prevalence ≥0.1% | Viral infections | Acute or chronic hepatitis B virus infection |
| Indicator conditions with expected undiagnosed HIV prevalence ≥0.1% | Viral infections | Acute or chronic hepatitis C virus infection |
| Indicator conditions with expected undiagnosed HIV prevalence ≥0.1% | Viral infections | Mononucleosis-like illness |
| Additional nationally recommended indications | Viral infections | Hepatitis A |
| AIDS-defining conditions | Parasitic infections | Cerebral toxoplasmosis |
| AIDS-defining conditions | Parasitic infections | Cryptosporidiosis lasting >1 month |
| AIDS-defining conditions | Parasitic infections | Isosporiasis lasting >1 month |
| Indicator conditions with expected undiagnosed HIV prevalence ≥0.1% | Parasitic infections | Visceral leishmaniasis |
| Additional nationally recommended indications | Parasitic infections | Atypical disseminated leishmaniasis |
| Additional nationally recommended indications | Parasitic infections | Reactivation of American trypanosomiasis with meningoencephalitis or myocarditis |
| AIDS-defining conditions | Bacterial infections | Mycobacterium tuberculosis, pulmonary or extrapulmonary |
| AIDS-defining conditions | Bacterial infections | Disseminated or extrapulmonary Mycobacterium avium complex or Mycobacterium kansasii infection |
| AIDS-defining conditions | Bacterial infections | Extrapulmonary or disseminated infection caused by other Mycobacterium species |
| AIDS-defining conditions | Bacterial infections | Recurrent pneumonia |
| AIDS-defining conditions | Bacterial infections | Recurrent Salmonella septicaemia |
| Indicator conditions with expected undiagnosed HIV prevalence ≥0.1% | Bacterial infections | Severe bacterial pneumonia |
| Indicator conditions with expected undiagnosed HIV prevalence ≥0.1% | Bacterial infections | Invasive pneumococcal disease |
| AIDS-defining conditions | Fungal infections | Pneumocystis jirovecii pneumonia |
| AIDS-defining conditions | Fungal infections | Esophageal candidiasis |
| AIDS-defining conditions | Fungal infections | Candidiasis of bronchi, trachea, or lungs |
| AIDS-defining conditions | Fungal infections | Extrapulmonary cryptococcosis |
| AIDS-defining conditions | Fungal infections | Disseminated or extrapulmonary histoplasmosis |
| AIDS-defining conditions | Fungal infections | Disseminated or extrapulmonary coccidioidomycosis |
| Additional nationally recommended indications | Fungal infections | Candidemia |
| Additional nationally recommended indications | Fungal infections | Other invasive or clinically significant candidiasis |
| Additional nationally recommended indications | Fungal infections | Disseminated talaromycosis/penicilliosis |
| Indicator conditions with expected undiagnosed HIV prevalence ≥0.1% | Sexually transmitted infections and related conditions | Syphilis |
| Indicator conditions with expected undiagnosed HIV prevalence ≥0.1% | Sexually transmitted infections and related conditions | Gonorrhoea |
| Indicator conditions with expected undiagnosed HIV prevalence ≥0.1% | Sexually transmitted infections and related conditions | Chlamydia infection |
| Indicator conditions with expected undiagnosed HIV prevalence ≥0.1% | Sexually transmitted infections and related conditions | HPV-associated intraepithelial neoplasia |
| Indicator conditions with expected undiagnosed HIV prevalence ≥0.1% | Sexually transmitted infections and related conditions | Anogenital warts |
| Additional nationally recommended indications | Sexually transmitted infections and related conditions | Mpox |
| Indicator conditions with expected undiagnosed HIV prevalence ≥0.1% | Hematological conditions | Unexplained leukopenia or thrombocytopenia lasting ≥1 month |
| Indicator conditions with expected undiagnosed HIV prevalence ≥0.1% | Hematological conditions | Idiopathic or thrombotic thrombocytopenic purpura |
| AIDS-defining conditions | Oral conditions | Oral hairy leukoplakia |
| Additional nationally recommended indications | Dermatological conditions | Seborrheic dermatitis or exanthem |
| Additional nationally recommended indications | Dermatological conditions | Severe or atypical psoriasis |
| Additional nationally recommended indications | Dermatological conditions | Molluscum contagiosum |
| Additional nationally recommended indications | Neurological conditions | Lymphocytic meningitis |
| Additional nationally recommended indications | Neurological conditions | Guillain-Barré syndrome |
| Additional nationally recommended indications | Neurological conditions | Mononeuritis multiplex |
| Additional nationally recommended indications | Neurological conditions | Subcortical dementia |
| Additional nationally recommended indications | Neurological conditions | Multiple sclerosis-like disease |
| Additional nationally recommended indications | Neurological conditions | Peripheral neuropathy |
| Indicator conditions with expected undiagnosed HIV prevalence ≥0.1% | Unexplained systemic or clinical conditions | Unexplained fever |
| Indicator conditions with expected undiagnosed HIV prevalence ≥0.1% | Unexplained systemic or clinical conditions | Unexplained weight loss |
| Indicator conditions with expected undiagnosed HIV prevalence ≥0.1% | Unexplained systemic or clinical conditions | Unexplained lymphadenopathy |
| Indicator conditions with expected undiagnosed HIV prevalence ≥0.1% | Unexplained systemic or clinical conditions | Unexplained oral candidiasis |
| Indicator conditions with expected undiagnosed HIV prevalence ≥0.1% | Unexplained systemic or clinical conditions | Unexplained chronic diarrhoea |
| Additional nationally recommended indications | Unexplained systemic or clinical conditions | Unexplained chronic renal failure |
| Additional nationally recommended indications | Central nervous system lesions | Primary space-occupying lesion of the brain |
| Additional nationally recommended indications | Pregnancy | Pregnancy |

The table presents the complete list of HIV testing indications used in the HIV-ICs-TR point-prevalence survey. The study framework combined international indicator-condition-guided HIV testing guidance, CDC AIDS-defining conditions, conditions in which unrecognised HIV infection could substantially affect clinical management, and the nationally recommended indicator-disease list circulated by the Republic of Türkiye Ministry of Health, General Directorate of Public Health, in 2025. The Ministry document was an official institutional communication distributed to healthcare institutions and was not included as a conventional publicly accessible reference in the manuscript. Conditions in which unrecognised HIV could affect clinical management correspond to the adverse-outcome indicator-condition category used in patient-level analyses. Categories describe the analytic framework, and some clinical conditions may overlap conceptually across international and national guidance frameworks.

Abbreviations: CDC, Centers for Disease Control and Prevention; HIV, human immunodeficiency virus; HPV, human papillomavirus.
