# Supplementary material for: Missed Opportunities for HIV Testing in Hospitalised Adults in Türkiye: Indicator Conditions and Testing Coverage in a National Multicentre Point-Prevalence Survey (HIV-ICs-TR)
Source: Sci Rep. 2026 May 30;16:23715. doi: 10.1038/s41598-026-54294-6 (PMC13427839; doi:10.1038/s41598-026-54294-6)
Supplement: Supplementary file 4 — Supplementary Information. [file 41598_2026_54294_MOESM4_ESM.docx]

Supplementary Table S4 Clinical characteristics and reasons for residual missed HIV testing among patients with documented testing indications

| **Age** | **Sex** | **Admitting department** | **Testing indication** | **Self-reported HIV exposure risk** | **Reason for missed testing** |
| --- | --- | --- | --- | --- | --- |
| 51 | Male | ICU | Pneumocystis jirovecii pneumonia | None reported | No study-team recommendation documented |
| 68 | Male | ICU | Severe bacterial pneumonia | None reported | Patient declined HIV testing (imminent discharge) |
| 70 | Female | ICU | Autoimmune disease receiving immunosuppressive therapy | None reported | Patient declined HIV testing (imminent discharge) |
| 78 | Male | ICU | Severe bacterial pneumonia | None reported | Patient declined HIV testing (imminent discharge) |
| 86 | Female | ICU | HBV infection | None reported | Primary clinical team declined testing recommendation |
| 33 | Male | Internal medicine | Autoimmune disease receiving immunosuppressive therapy | None reported | No study-team recommendation documented |
| 46 | Male | Internal medicine | Solid malignancies receiving chemotherapy | None reported | No study-team recommendation documented |
| 53 | Male | Internal medicine | Sexually transmitted infection | Reported STI history | Patient declined HIV testing (imminent discharge) |
| 57 | Female | Internal medicine | Herpes zoster | Reported STI history | Patient declined HIV testing (self-perceived low risk) |
| 61 | Male | Internal medicine | Esophageal candidiasis | None reported | Patient declined HIV testing (self-perceived low risk) |
| 62 | Female | Internal medicine | Severe bacterial pneumonia | None reported | Patient declined HIV testing (self-perceived low risk) |
| 62 | Male | Internal medicine | Unexplained weight loss | None reported | Patient declined HIV testing (imminent discharge) |
| 65 | Female | Internal medicine | Severe bacterial pneumonia | None reported | Patient declined HIV testing (imminent discharge) |
| 65 | Male | Internal medicine | Severe bacterial pneumonia | None reported | Patient declined HIV testing (self-perceived low risk) |
| 67 | Female | Internal medicine | Severe bacterial pneumonia | None reported | Patient declined HIV testing (imminent discharge) |
| 71 | Female | Internal medicine | Severe bacterial pneumonia | None reported | Patient declined HIV testing (imminent discharge) |
| 71 | Female | Internal medicine | Severe bacterial pneumonia | None reported | Patient declined HIV testing (self-perceived low risk) |
| 91 | Female | Internal medicine | Severe bacterial pneumonia | None reported | Patient declined HIV testing (self-perceived low risk) |
| 33 | Male | Surgical | HBV infection | Reported STI history | Patient declined HIV testing (imminent discharge) |
| 52 | Female | Surgical | Solid malignancies receiving chemotherapy | None reported | No study-team recommendation documented |
| 65 | Male | Surgical | High-grade anal dysplasia / anal cancer | None reported | No study-team recommendation documented |
| 73 | Male | Surgical | Severe bacterial pneumonia | None reported | Patient declined HIV testing (imminent discharge) |
| **Note:** This table includes patients with documented HIV testing indications in whom HIV testing was not completed after the index-day assessment workflow. “None reported” indicates no self-reported HIV exposure risk documented during the assessment. “No study-team recommendation documented” indicates that HIV testing was not actively recommended by the study team during the survey workflow despite a documented testing indication. Reasons for missed testing were abstracted from the study assessment forms.  **Abbreviations:** HBV, hepatitis B virus; HIV, human immunodeficiency virus; ICU, intensive care unit; STI, sexually transmitted infection. | | | | | |
