# Supplementary material for: Missed Opportunities for HIV Testing in Hospitalised Adults in Türkiye: Indicator Conditions and Testing Coverage in a National Multicentre Point-Prevalence Survey (HIV-ICs-TR)
Source: Sci Rep. 2026 May 30;16:23715. doi: 10.1038/s41598-026-54294-6 (PMC13427839; doi:10.1038/s41598-026-54294-6)
Supplement: Supplementary file 5 — Supplementary Information. [file 41598_2026_54294_MOESM5_ESM.docx]

**Supplementary Table S5** Clinical characteristics and prior HIV testing indications among hospitalised people living with HIV (n = 15)

| **Patient** | **Age (years)** | **Sex** | **CD4 cell count at HIV diagnosis (cells/mm³)** | **Documented condition or indication preceding HIV diagnosis** | **Interval from indication/presentation to HIV diagnosis, months** | **HIV testing performed at initial presentation** |
| --- | --- | --- | --- | --- | --- | --- |
| Patient 1 | 37 | Male | 64 | Herpes zoster | 7 | No |
| Patient 2 | 42 | Male | 378 | None | — | — |
| Patient 3 | 53 | Male | 402 | None | — | — |
| Patient 4 | 22 | Male | 157 | Cryptococcal meningitis | 1 | Yes |
| Patient 5 | 33 | Male | 89 | Unexplained cognitive impairment | 11 | No |
| Patient 6 | 46 | Male | 44 | Pneumocystis jirovecii pneumonia | 1 | Yes |
| Patient 7 | 45 | Male | 141 | None | — | — |
| Patient 8 | 33 | Male | 65 | Genitourinary tuberculosis | 1 | Yes |
| Patient 9 | 21 | Female | 250 | None | — | — |
| Patient 10 | 32 | Female | 550 | None | — | — |
| Patient 11 | 64 | Female | 202 | None | — | — |
| Patient 12 | 42 | Female | 90 | None | — | — |
| Patient 13 | 46 | Male | 96 | Pulmonary tuberculosis | 1 | Yes |
| Patient 14 | 52 | Male | 258 | None | — | — |
| Patient 15 | 28 | Male | 10 | None | — | — |

**Note:** All individuals listed in this table had a previously known HIV diagnosis at the time of the index-day assessment and were excluded from the main analysis of hospitalised adults without known HIV infection. “—” indicates not applicable because no prior AIDS-defining condition or HIV testing indication was documented before HIV diagnosis.

**Abbreviations:** AIDS, acquired immunodeficiency syndrome; CD4, CD4+ T-cell count; HIV, human immunodeficiency virus; PLWH, people living with HIV.
